# Evaluation of renal microperfusion in hyperuricemic nephropathy by contrast-enhanced ultrasound imaging

**DOI:** 10.1242/dmm.049382

**Published:** 2022-07-26

**Authors:** Li He, Ze Li, Qunzi Zhang, Yini Chen, Yihui Gao, Teng Chen, Niansong Wang, Lixin Jiang, Ying Fan

**Affiliations:** 1 Department of Nephrology, Shanghai Jiao Tong University Affiliated Sixth People's Hospital, Shanghai 200233, China; 2 Ultrasonic Department, Shanghai Jiao Tong University Affiliated Sixth People's Hospital, Shanghai 200233, China; 3 Ultrasonic Department, Renji Hospital, School of Medicine, Shanghai Jiao Tong University, Shanghai 200127, China

**Keywords:** Hyperuricemic nephropathy, Hyperuricemia, CEUS, Renal cortical perfusion, Peak intensity

## Abstract

Diagnostic tools for the early detection of renal injury caused by hyperuricemia are still lacking. Here, we investigated whether contrast-enhanced ultrasound (CEUS) could be used as a diagnostic tool for hyperuricemic nephropathy (HN). In the HN rat model, CEUS detected a significant decline in renal cortical perfusion compared with that in control rats. Peak intensity (PI) values correlated significantly with serum KIM-1 levels and fibrosis scores in HN rats. An early decline in PI values was also observed in chronic kidney disease (CKD) stage 1 patients with HN compared with the controls (61.1±4.52 dB versus 65.80±7.10 dB) and correlated with renal function in the patients with HN. In contrast, an increase in time to reach PI values was detected in HN patients with stage 1 CKD (15.14±1.75 s versus 14.52±4.75 s) and was more pronounced in CKD stage 4 patients (67.32±3.29 s). CEUS was able to detect abnormal renal perfusion in early CKD with HN, which correlated with renal function decline, suggesting that CEUS could be used as a noninvasive tool for assessing renal function in patients with HN.

## INTRODUCTION

Hyperuricemia plays a causal role in the development of metabolic syndrome, cardiovascular diseases and renal diseases (Dehlin et al., 2020; De Vera et al., 2010; Kuo et al., 2013; Yu et al., 2012). Increasing evidence indicates that hyperuricemia is an independent risk marker for the progression of chronic kidney disease (CKD) (He et al., 2017). However, the early effect of hyperuricemia on renal injury and its dynamic changes in physiological pathology during the course of disease are poorly understood. Studies have indicated that uric acid can induce direct obstruction in tubules, resulting in renal tubular cell injury and subsequent interstitial fibrosis (Sánchez-Lozada et al., 2007; Mazzali et al., 2001). This finding was confirmed by the deposition of uric acid crystals surrounded by monocytes or macrophages in the kidneys of hyperuricemic nephropathy (HN) rats (Mazzali et al., 2001). The histologic injury termed HN consists of renal arteriolosclerosis, glomerulosclerosis and interstitial fibrosis, which is accompanied by focal urate crystal deposition. The same pathological findings have been found in autopsies of 79-99% of patients with gout (Kang et al., 2002). However, there is still no consensus or guidelines regarding the diagnostic criteria for HN. Currently, the diagnosis of HN is mainly based on medical history, clinical manifestations and kidney function, and the results of routine urinalysis are usually not specific with trace proteinuria or hematuria. Therefore, it is essential to develop more tools to detect hyperuricemia-induced kidney injury in the early stage.

Over 90% of kidney blood flow in the kidney cortex is provided by capillaries and small kidney arterioles (Ma et al., 2012). Downregulation of renal blood flow is believed to play a key role in the pathogenesis of CKD (Schneider et al., 2013a,b; Schrier and Wang, 2004). It is conceivable that serum uric acid may be associated with the regulation of renal perfusion (Wang et al., 2015). Variations in blood flow within the medulla or the cortex likely occur in HN. Thus, early detection of renal microvascular perfusion abnormalities should be a reasonable approach to aid in the diagnosis of HN in the early stage.

Conventional ultrasonography has been widely applied in the evaluation of kidney structure as well as basic function for more than 40 years in routine clinical practice. Ultrasonography began with grayscale ultrasound and rapidly developed into color (CDUS) and power (PDUS) Doppler ultrasound and pulsed wave Doppler (PWD) (Correas et al., 2016). These techniques are able to detect blood flow disturbances in the kidney. However, poor performance in recognizing low-speed flow signals and angle dependency remains a major limitation (Yongfeng et al., 2016). Contrast-enhanced ultrasound (CEUS) is a new imaging technique using microbubble-based contrast agents and low mechanical index ultrasonography that can detect slower blood flow in smaller blood vessels than CDUS can (Wang and Mohan, 2016). Recent studies have shown that CEUS can be used as a sensitive diagnostic tool for measuring renal perfusion in kidney diseases with no renal toxicity (Correas et al., 2016; Kalantarinia et al., 2009; Schneider et al., 2012, 2014, 2015). Because more than 90% of the total renal blood flow enters the renal cortex, CEUS is regarded as the perfect tool for detecting renal parenchymal perfusion in real time (Granata et al., 2011). For example, CEUS was used to measure renal perfusion in both rats and humans with acute kidney injury (AKI) and could serve as a noninvasive method to assess AKI severity and predict its progression to CKD (Cao et al., 2017).

However, the role of CEUS in HN has not been studied. Therefore, the present study was designed to assess whether CEUS could be used as a sensitive and noninvasive method of measuring renal microvascular perfusion changes and evaluating disease severity and progression in hyperuricemia-related kidney injury.

## RESULTS

### Renal function decline and renal tubular interstitial injury correlate with disease severity in rats with HN

To examine hyperuricemia-induced kidney injury in the animal model, rats were gavaged with adenine (0.1 g/kg) and potassium oxonate (1.5 g/kg) daily for 4 weeks. One week after administration of adenine and potassium oxonate, serum uric acid (SUA), serum creatinine (SCr), blood urea nitrogen (BUN), urine albumin/creatinine ratio (UACR) and the kidney/body weight ratio were significantly increased, and all these parameters reached their highest levels at 4 weeks after adenine and potassium oxonate administration in hyperuricemic rats compared with the control rats (Table S1). These data suggest that these rats developed hyperuricemia-induced kidney injury and could be used as a model for HN.

**Fig. 1.**
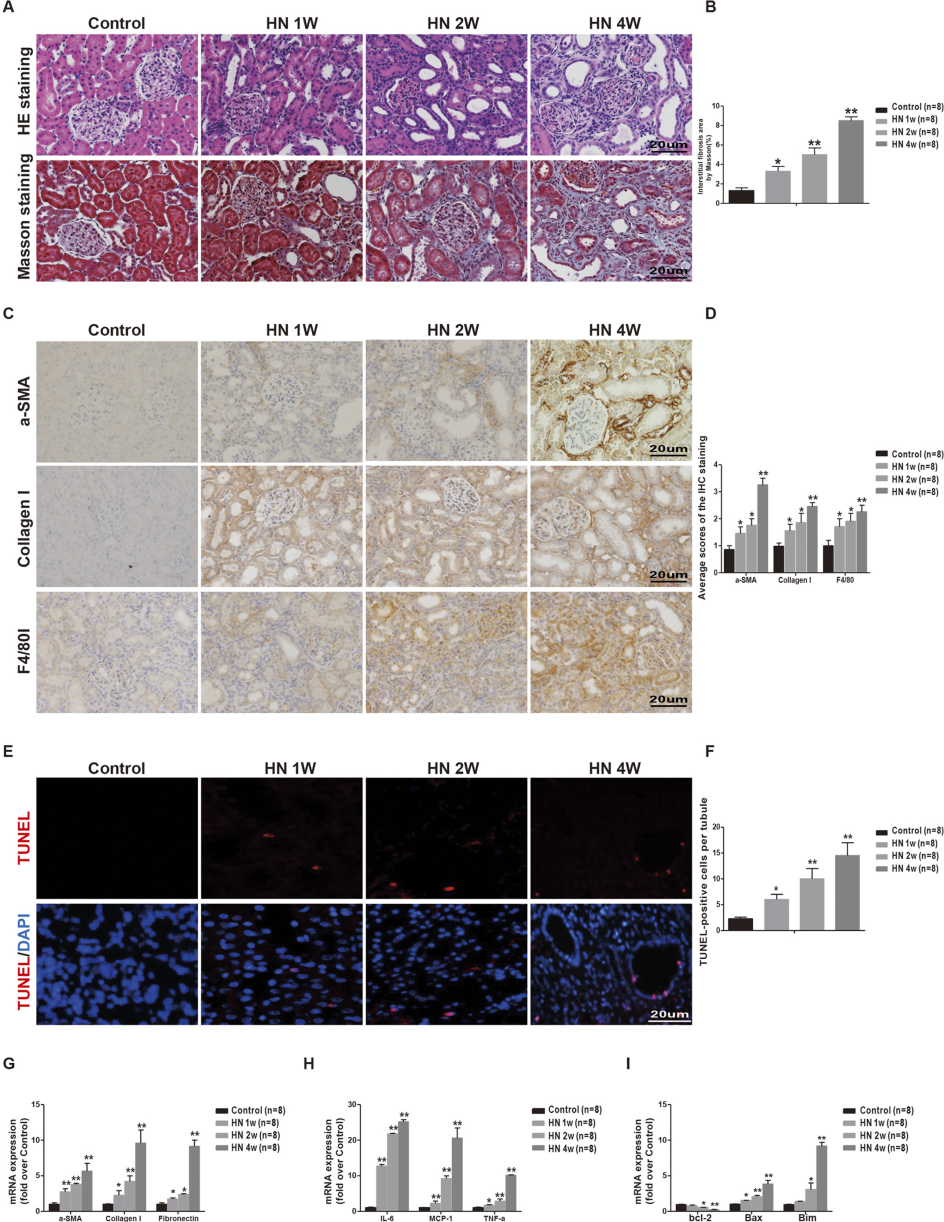
**Renal dysfunction and pathological injury of the renal tubular interstitium correlates with disease severity in hyperuricemic rats.** (A) H&E and Masson staining of kidney sections were compared between different groups of rats (original magnification 400×). (B) Semiquantitative analysis of renal interstitial fibrosis area in kidney sections. (C) Immunohistochemistry staining for collagen 1, α-SMA and F4/80 in the kidneys; representative pictures are shown (original magnification 400×). (D) Semiquantitative data of collagen 1, α-SMA and F4/80 staining in different groups of rats. IHC, immunohistochemical. (E) TUNEL staining to measure apoptosis of renal tubular epithelial cells in each rat group (original magnification 400×). (F) Quantitative analysis of the number of apoptotic cells in each rat group. (G) Real-time PCR analyses of genes encoding inflammatory markers. (H) The mRNA expression of inflammatory markers in different groups of rats. (I) Apoptosis-related markers were detected by real-time PCR. Data are presented as the mean±s.e.m. of four experiments. *n*=8; *P<0.05, **P<0.01 versus control group (unpaired, two-tailed *t*-test). Scale bars: 20μm.

We then performed histological tests to examine the pathological changes in HN rat kidneys. Hematoxylin and Eosin (H&E) and Masson's trichrome staining showed that at 1 week after administration of adenine and potassium oxonate, hyperuricemic rats began to develop tubular dilatation and sloughing of tubular epithelial cells or loss of the brush border (Fig. 1A,B). The rats showed significant kidney fibrosis and increased focal infiltration of inflammatory cells at 4 weeks after establishment of the model, as shown by real-time PCR of inflammatory markers and immunostaining for α-SMA (also known as ACTA2), collagen 1 and F4/80 (also known as ADGRE1) in the kidney (Fig. 1C,D,G,H). In addition, renal tubular cell apoptosis was shown by terminal deoxynucleotidyl transferase dUTP nick end labeling (TUNEL) staining, with the upregulation of proapoptotic genes *Bim* (also known as *Bcl2l11*) and *Bax2*, and the downregulation of the antiapoptotic gene *Bcl2*, in the kidneys of HN rats at 4 weeks (Fig. 1E,F,I). Our data suggest that tubular cell injury, kidney interstitial fibrosis and inflammation were all present in this hyperuricemic rat model.

### Monosodium urate crystal deposits in the kidneys of HN rats

**Fig. 2.**
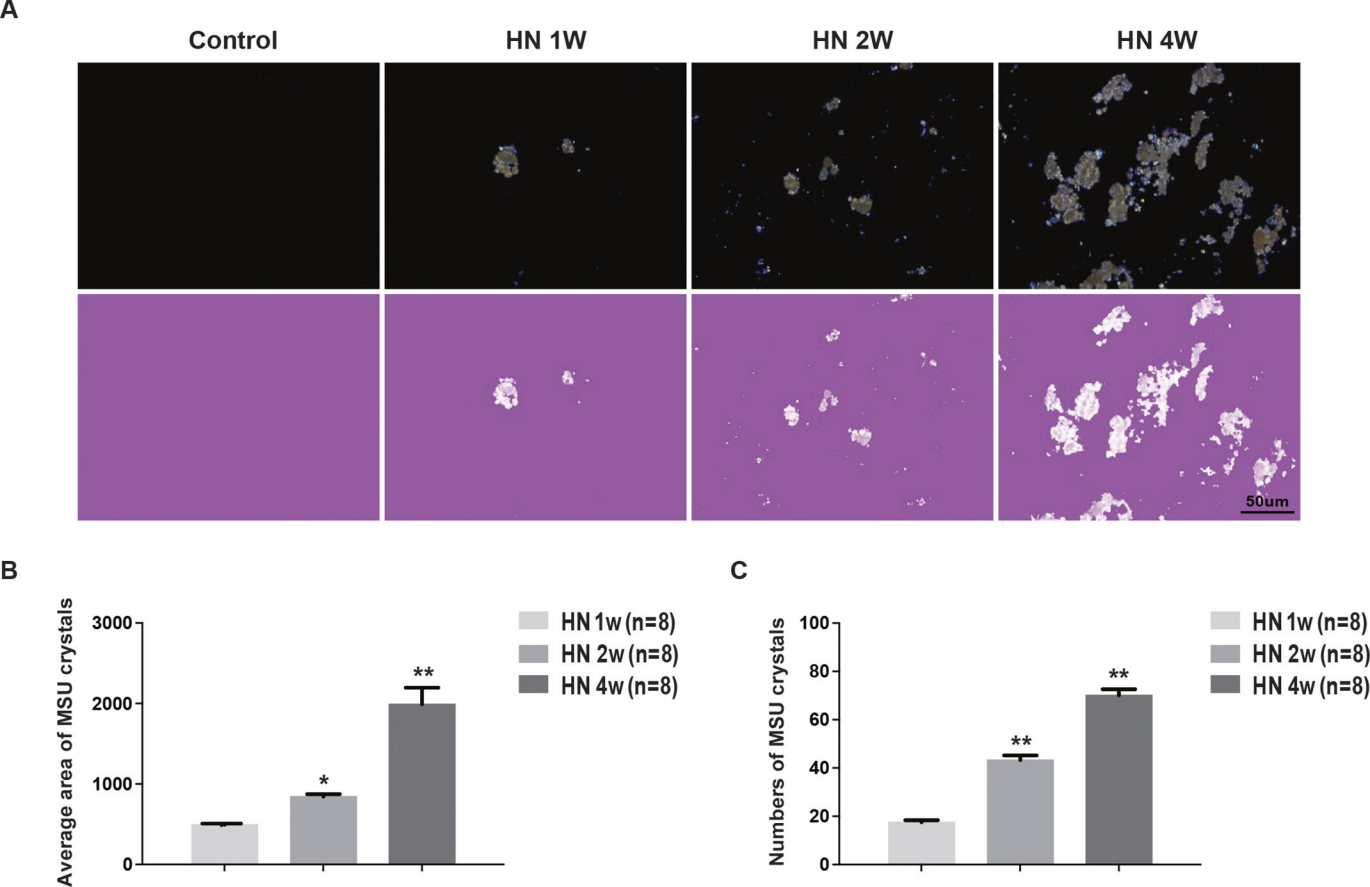
**Monosodium urate (MSU) crystal deposits in the kidneys of hyperuricemic rats.** (A) Kidney sections under compensated polarized light showing anisotropic uric acid crystals in the kidney (original magnification 200×). Scale bar: 50 μm. (B) Semiquantitative analysis of urate crystal areas in kidney sections. (C) Semiquantitative analysis of urate crystal numbers in kidney sections. Data are presented as the mean±s.e.m. of four experiments. *n*=8; *P<0.05, **P<0.01 versus control group (unpaired, two-tailed *t*-test). HN, hyperuricemic nephropathy.

Because hyperuricemia contributes to the deposition of monosodium urate crystals (MUCs) in tissues, we used a compensated polarized light microscope to detect MUC deposition in the kidneys of HN rats. The data showed that at 1 week after administration of adenine and potassium oxonate, HN rats began to exhibit scattered crystals in the kidney, particularly in the tubular compartment, and the crystals increased in size and aggregated in all the major areas of the kidney at 4 weeks (Fig. 2A). Quantification data confirmed that the average number and area of MUCs in HN rat kidneys gradually increased with time (Fig. 2B,C). These data indicate that renal tubular injury, fibrosis and MUC deposition were all present in the HN rat kidneys.

### Renal blood flow measured by ultrasound in rats with mild and severe HN

**Fig. 3.**
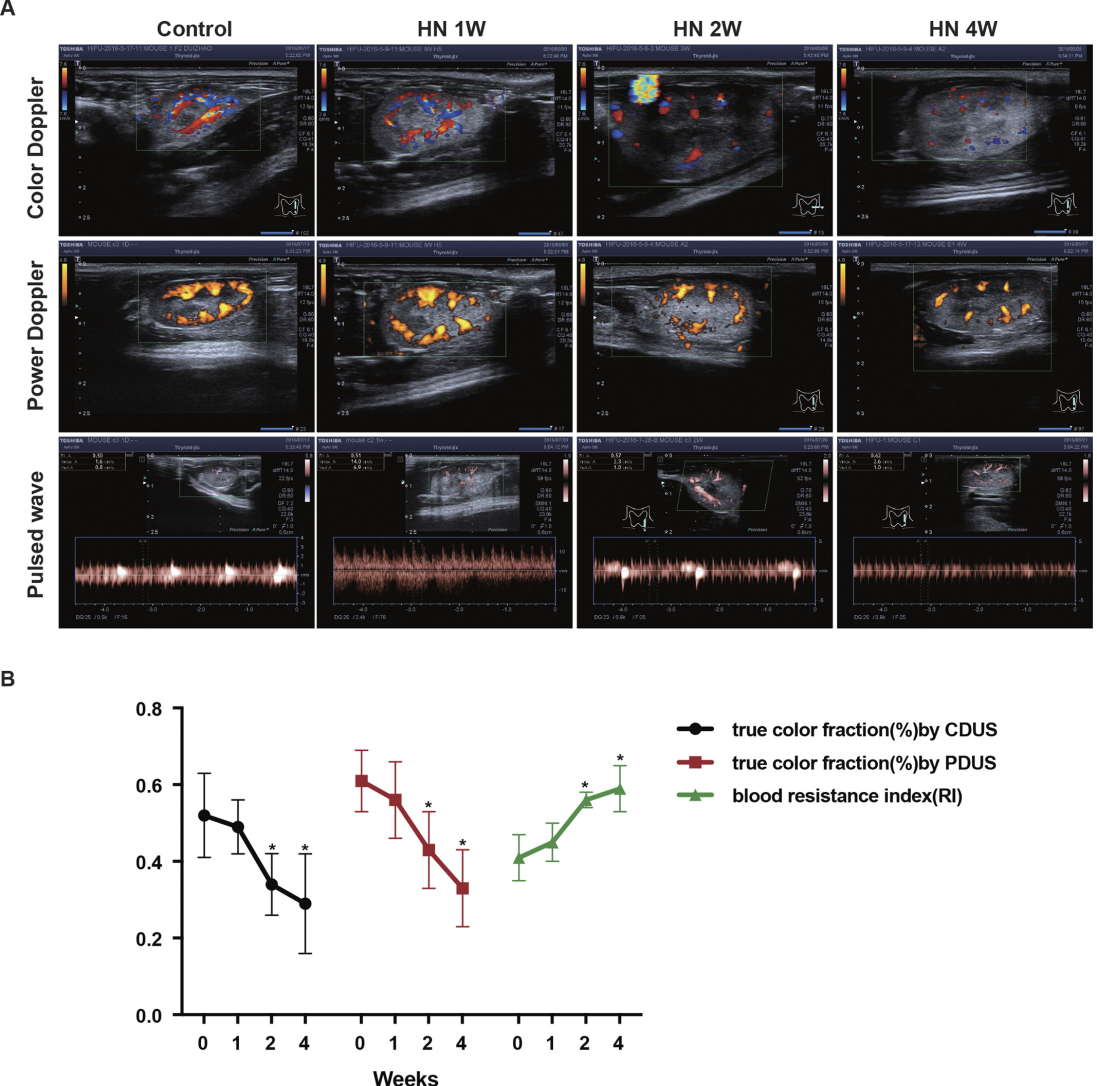
**Renal blood flow measured by ultrasound in rats with mild and severe HN.** (A) Ultrasound images. (B) Semiquantitative data of blood flow. *n*=8; *P<0.05 versus control group (unpaired, two-tailed *t*-test). CDUS, color Doppler ultrasound; PDUS, power Doppler ultrasound.

Ultrasound was performed to further assess the morphological and functional changes in the kidneys of HN rats. CDUS/PDUS showed that the blood flow in the kidneys began to decrease 2 weeks after administration of adenine and potassium oxonate and decreased further at 4 weeks after the establishment of the model (Fig. 3A). The area of colored pixels seen on the B-mode image was measured with ImageJ software for the quantification of the blood flow index measured by CDUS/PDUS. The data showed that the percentage area of colored pixels at 1 week after adenine and potassium oxonate administration was not significantly different from that of normal rats (0.49±0.07/0.56±0.10 versus 0.52±0.11/0.61±0.08). However, the percentage area of colored pixels began to decrease in the second week (0.34±0.08/0.43±0.10) and reached the lowest level in the fourth week (0.29±0.13/0.33±0.10). In addition, PWD showed that the vascular resistance index (RI) value of HN rat kidneys began to increase at 2 weeks (0.56±0.02) compared with that of the normal control kidneys (0.41±0.06) (Fig. 3B). However, neither CDUS/PDUS nor PWD could detect earlier changes in renal blood flow at 1 week after the HN model was established.

### Dynamic assessment of renal cortical perfusion in the HN rat model by CEUS

**Fig. 4.**
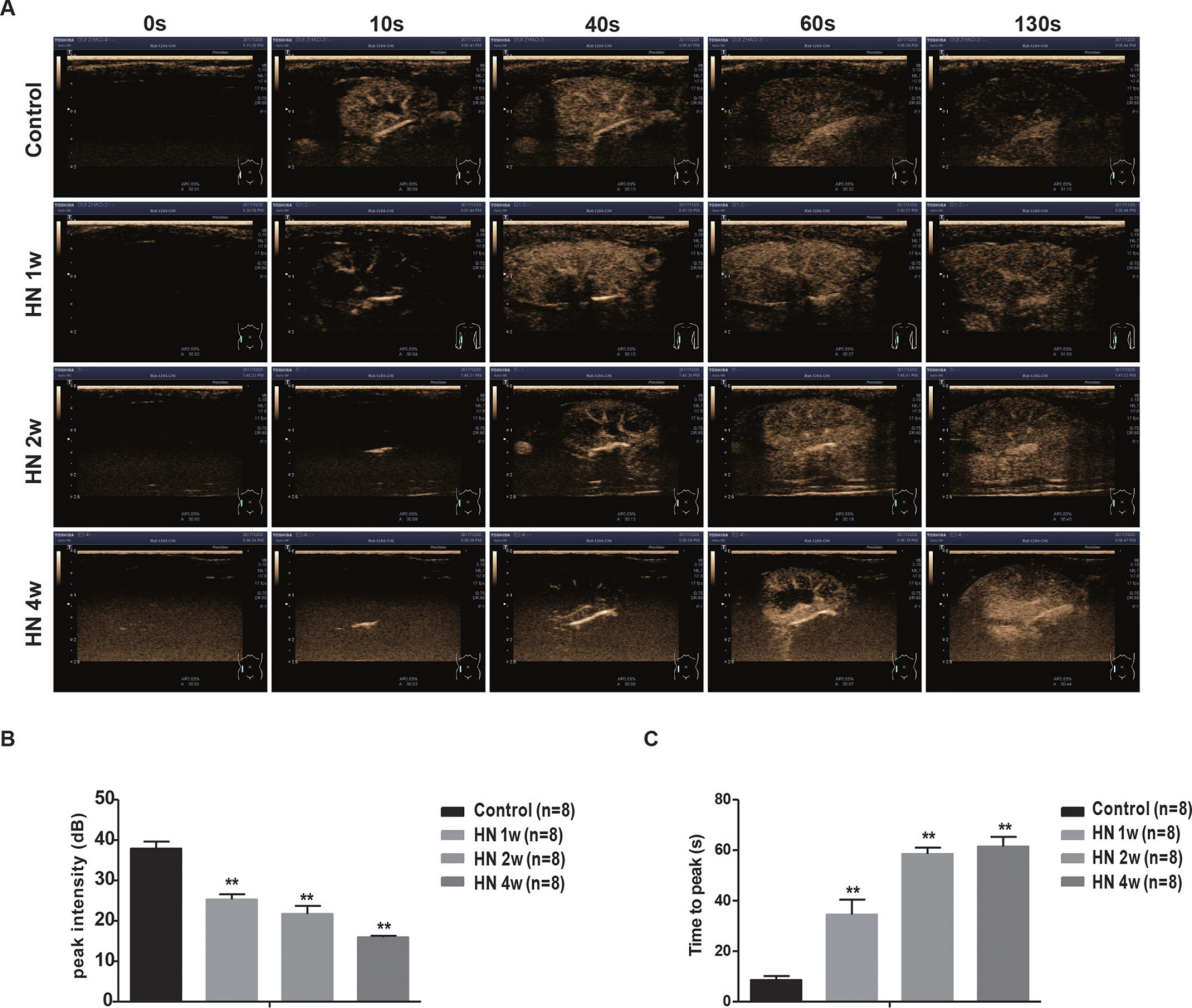
**Dynamic assessment of renal cortical perfusion in the hyperuricemic rat model by contrast-enhanced ultrasound (CEUS).** (A) Ultrasound images. (B) Peak intensity (PI) values. (C) Time to reach peak intensity (TTP) values. *n*=8; **P<0.01 versus control group (unpaired, two-tailed *t*-test).

We then applied the CEUS technique to monitor the effects of uric acid on renal microvascular perfusion in HN rats. Microbubble contrast agents exhibited a triphasic enhancement pattern within the intact kidney. Enhancement of interlobular arteries and the renal artery was observed first, followed by enhancement of the renal cortex and renal medulla. All three phases were detected in the HN rat model with the imaging system described here. All rats were imaged through three stages: ‘start to enhance’, ‘cortical peak’ and ‘wash-out phase’. The HN rats reached their cortical peak stage more slowly than normal controls, and they also took a longer time to progress into the wash-out phase, especially at 4 weeks after model creation (Fig. 4A).

Time-intensity curves (TICs) in the renal cortex were acquired for all rats. After measurement with SonoLiver software, quantitative perfusion indexes of CEUS were accurately obtained for all rats and selected for final data analysis. The TICs of renal cortex perfusion were asymmetrical curves and included three parts: steep ascending slope, peak and flat descending slope. As shown in Fig. 4B,C, a significant decline in renal cortical perfusion, as reflected by a lower peak intensity (PI) value and longer time to reach peak intensity (TTP), was observed in HN rats compared with control rats (PI, 25.43±1.31 dB versus 37.9±1.75 dB; TTP, 34.5±5.9 s versus 8.58±1.6 s) at 1 week after administration of adenine and potassium oxonate, with a more pronounced decline at 4 weeks (PI, 15.93±0.40 dB; TTP, 61.4±3.9 s). Therefore, by evaluating renal microvascular perfusion, CEUS can detect early renal dysfunction in HN rats.

### PI measured by CEUS correlates with renal tubulointerstitial injury in HN rats

**Fig. 5.**
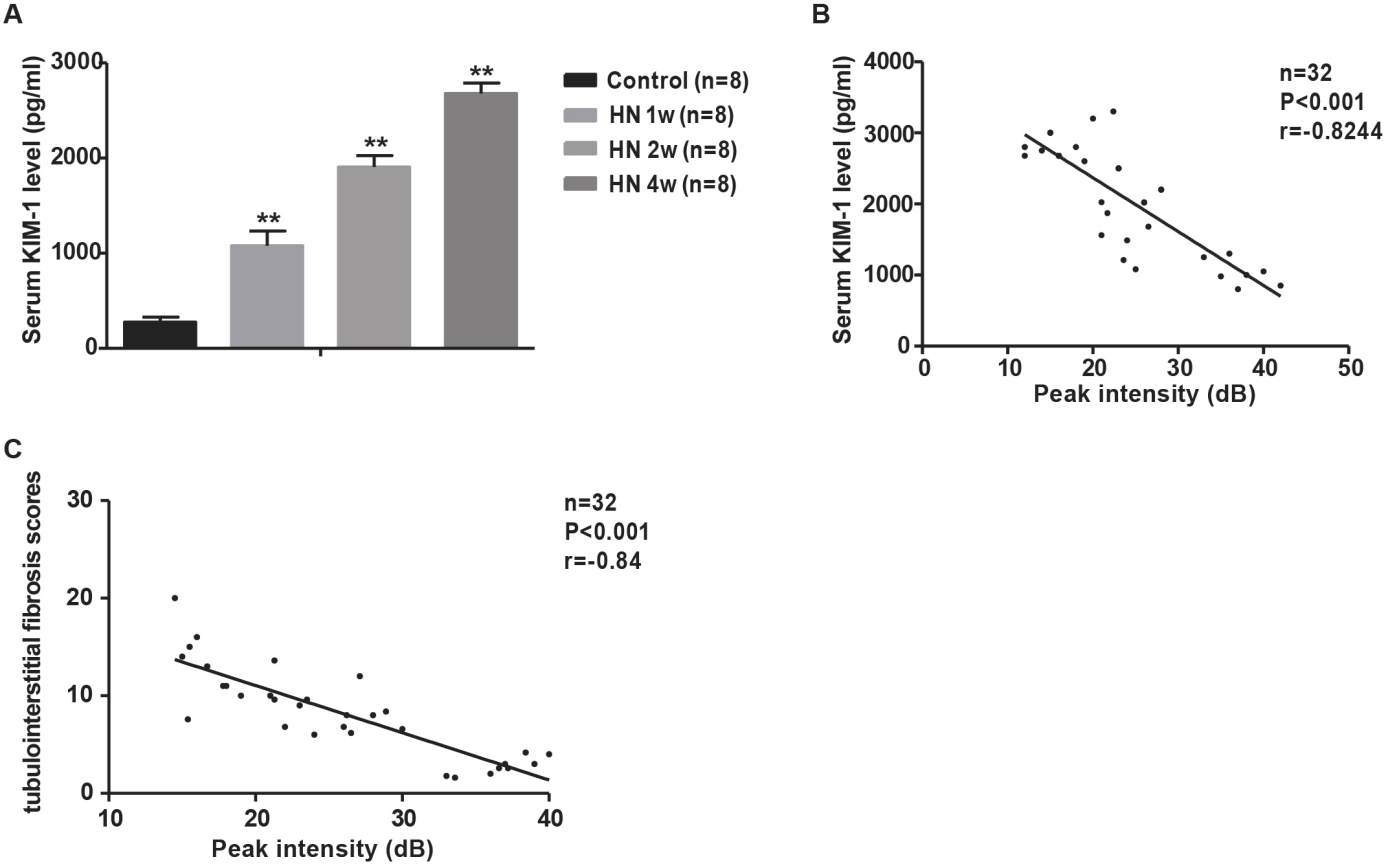
**PI as measured by CEUS correlates with renal tubulointerstitial injury in hyperuricemic rats.** (A) Serum KIM-1 levels were examined with a KIM-1 ELISA kit. (B) Renal cortical perfusion was correlated with serum KIM-1 levels. (C) Renal cortical perfusion was correlated with fibrosis scores. Data are presented as the mean±s.e.m. of four groups. *n*=8; **P<0.01 versus control group (unpaired, two-tailed *t*-test).

Our data suggest that CEUS can detect an early decline in renal microvascular perfusion in our HN rat model. Next, we assessed whether the quantitative index of CEUS correlated with renal injury and function in HN rats. We measured serum KIM-1 (also known as HAVCR1), a well-known tubular injury marker, and quantified kidney fibrosis scores in HN rats at various disease stages. As shown in Fig. 5A, the levels of serum KIM-1 were elevated at 1 week (1189.3±111.5 pg/ml) after administration of adenine and potassium oxonate and further increased at 4 weeks (2500.1±222.8 pg/ml), suggesting that renal dysfunction was aggravated during disease progression. The quantitative assessment of PI by CEUS showed a correlation with the serum KIM-1 levels and the fibrosis scores in HN rats with mild or severe disease (Fig. 5B,C). These data suggest that the quantitative values of PI measured by CEUS had a good correlation with renal function and tubulointerstitial injury in HN rats.

### CEUS evaluation in patients with HN

To further evaluate whether CEUS imaging can be applicable in clinical use, renal cortical perfusion was measured by CEUS in 40 patients who were admitted for hyperuricemia-induced kidney injury (HN) in different CKD stages. Each CKD stage included ten patients, and ten age- and sex-matched healthy volunteers were used as controls. All the patients had more than a 5-year history of hyperuricemia, recurrent attacks of gout and kidney stones detected by ultrasound. The characteristics of the HN patients are presented in Table 1. The patients with HN had higher serum levels of blood urea nitrogen (BUN) and creatinine, a longer disease duration and more frequent gout attacks than the healthy controls.

**Table 1.**
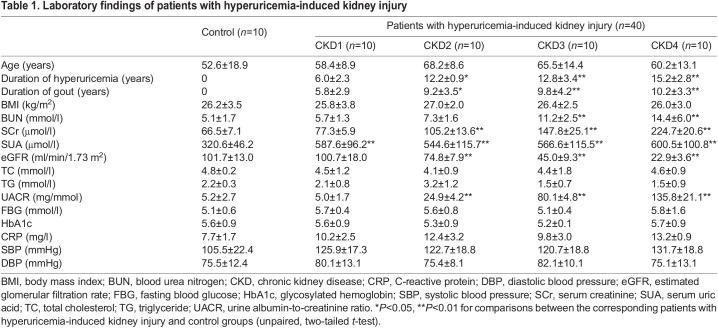
Laboratory findings of patients with hyperuricemia-induced kidney injury

**Fig. 6.**
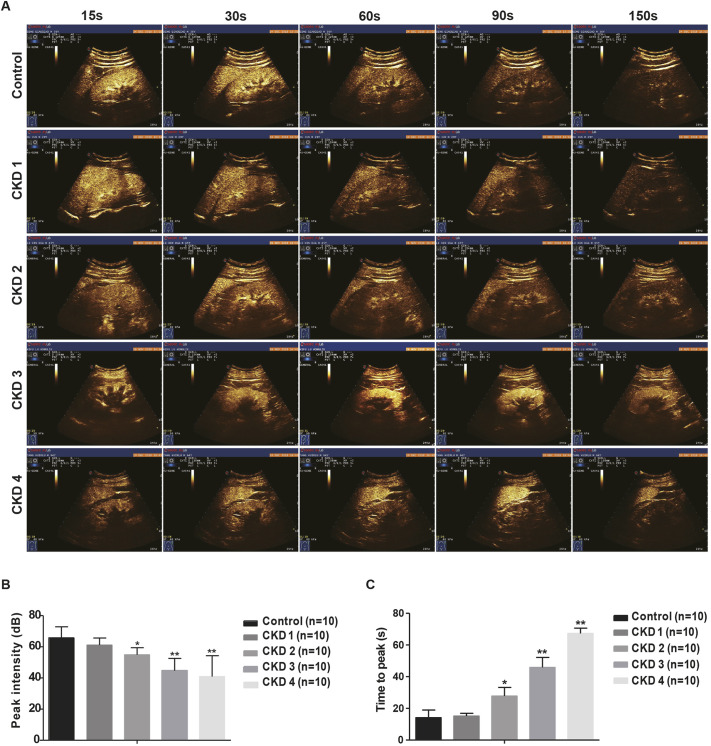
**CEUS evaluation of renal perfusion in hyperuricemia-induced kidney injury in humans.** (A) Representative serial contrast enhancement images in humans. (B) PI values of patients with HN and different chronic kidney disease (CKD) stages. (C) TTP values of patients with HN and different CKD stages. *P<0.05, **P<0.01 for comparisons between the corresponding HN and control groups (unpaired, two-tailed *t*-test).

CEUS was then performed in these patients. HN patients reach their cortical peak stages slower than normal controls did, and they also took a longer time to progress into the wash-out phase. The most prominent perfusion loss was shown in HN patients with CKD stage 4 (Fig. 6A). As shown in Fig. 6B,C, HN patients exhibited lower renal cortical perfusion than healthy controls. An earlier decline in renal cortical perfusion was found in patients with CKD stage 1 than in the control group (PI, 61.1±4.52 dB versus 65.80±7.10 dB), and perfusion became progressively less visible in patients with more severe kidney injury. Perfusion was lowest in HN patients with CKD stage 4 (PI, 40.93±13.36 dB). TTP began to increase in HN patients with CKD stage 1 compared with normal controls (TTP, 15.14±1.75 s versus 14.52±4.75 s) and was longest in CKD stage 4 patients (67.32±3.29 s). Our data suggest that, consistent with the animal data, CEUS is a sensitive method for detecting early reduction in renal microperfusion and assessing CKD progression in patients with HN.

### PI measured by CEUS correlates with renal function in patients with HN

**Fig. 7.**
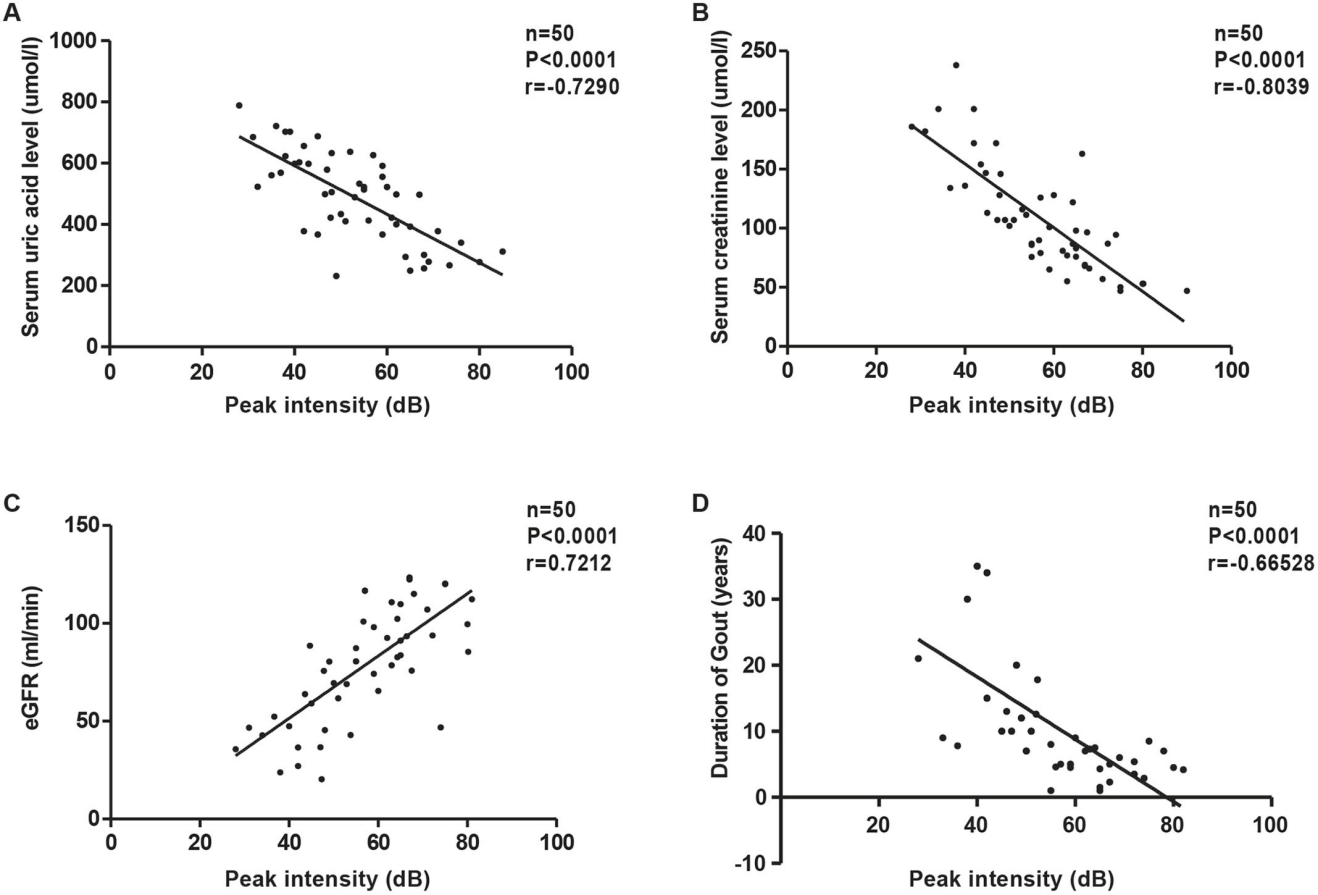
**PI measured by CEUS correlates with renal function in patients with hyperuricemia-induced kidney injury.** (A) PI correlated with the serum uric acid level. (B) PI correlated with the serum creatinine level. (C) PI correlated with the estimated glomerular filtration rate (eGFR) value. (D) PI correlated with the duration of gout. Unpaired, two-tailed *t*-test.

To examine the relationship between renal perfusion and the severity of kidney damage, we analyzed the correlation between the PI values measured by CEUS and renal function parameters. PI values were negatively correlated with serum creatinine and serum uric acid levels and positively correlated with the estimated glomerular filtration rate (eGFR) (Fig. 7A-C). We also found that the duration of gout history correlated with the PI value (Fig. 7D). Our data suggest that CEUS can be used not only to assess intrarenal blood flow but also to monitor the progression of renal function in HN patients.

## DISCUSSION

The prevalence of hyperuricemia and gout is increasing worldwide (Yu et al., 2018; Zhu et al., 2011). Uric acid is considered a potential risk factor for the development and progression of CKD (Sampson et al., 2017; Lu et al., 2018). Hyperuricemia causes kidney injury via the precipitation of urate crystals in the kidney, which results in renal tubular injury and interstitial fibrosis (Mazzali et al., 2001). Renal function usually declines early during disease progression without nephrotic proteinuria. To date, there are no specific biomarkers or methods for the early detection and diagnosis of HN. Therefore, it is important to develop a new method to improve the early detection and evaluation of HN disease severity.

In the present study, we report a dynamic change in hyperuricemia-induced kidney injury in a rat model of HN. We found that these rats exhibited progressive proteinuria and renal dysfunction from the first to fourth week in a time-dependent manner after daily administration of a mixture of adenine and potassium oxonate for 4 weeks. Histologically, we found progressive tubular damage, interstitial inflammation and fibrosis in the kidneys of HN rats. Deposition of monosodium urate crystals was detected as early as 1 week after the HN model was established, suggesting that hyperuricemia can lead to kidney injury at a very early stage.

To develop a noninvasive method for evaluating hyperuricemia-induced kidney injury *in vivo*, we applied CEUS in an HN rat model and compared this method with conventional ultrasonography methods, such as CDUS/PDUS and PWD. Although PWD was able to detect a decrease in renal blood flow during disease progression (Hull et al., 2017), CEUS was found to be more sensitive for detecting renal blood flow variation as early as 1 week after the administration of adenine and potassium oxonate. In addition, CEUS was able to collect real-time images of vascular perfusion, allowing continuous imaging of the vasculature and blood flow by using a microbubble contrast agent (Schneider et al., 2013b; Ting et al., 2012; Scheele et al., 1995; Fischer et al., 2016; Wang et al., 2017). Additionally, perfusion abnormalities such as PI or TTP significantly correlated with the severity of kidney injury, suggesting that CEUS was able to assess dynamic changes in renal perfusion impairment at an early disease stage in HN rats.

To our knowledge, this is the first study conducted in patients with HN using CEUS imaging to evaluate the renal injury caused by hyperuricemia. Higher uric acid levels correlate with a decreased glomerular filtration rate (GFR) (Johnson et al., 2018; Iseki et al., 2004, 2001; Weiner et al., 2008; Chonchol et al., 2007). Hyperuricemia may cause endothelial dysfunction, leading to a reduction in nitric oxide synthesis and upregulation of renal artery resistance (Hong et al., 2012; Khosla et al., 2005). *In vivo*, studies showed that high uric acid levels were associated with glomerular ischemia by reducing renal arteriole blood flow and increasing the resistance in the renal artery (Johnson et al., 2003; Zhou et al., 2006). Our data suggest that alterations in renal microvascular perfusion detected by CEUS could be early pathophysiological changes in patients with HN and correlated with renal function and tubular cell injury markers (KIM-1). Therefore, this noninvasive method could be used to help with the early detection and diagnosis of HN in clinical practice.

CEUS is currently regarded as a promising method for renal microvascular perfusion and kidney injury evaluation in several kidney diseases. For example, studies have shown that CEUS can be used to estimate renal microvascular perfusion in diabetic patients, cardiac surgery patients and AKI patients (Schneider et al., 2011; Ma et al., 2012). CEUS can also be used to predict acute kidney injury to chronic kidney disease progression in humans (Cao et al., 2017). Our findings suggest that CEUS has diagnostic value in the evaluation of renal perfusion in patients with HN. Compared with normal controls, patients with HN showed an obvious reduction in renal perfusion at an early stage, suggesting that the detection of renal perfusion may predict kidney injury in the early stage of HN. Early identification of patients who develop hyperuricemia-induced kidney injury would assist physicians in initiating the appropriate measures to protect the kidney by providing renal-preserving treatments. Our study also showed that the contrast agents for CEUS are safe in CKD patients.

This study has several limitations. First, CEUS assessments can be affected by several factors, such as the bubble properties of contrast agents, patient factors, scanner facilities and operators (Harrois and Duranteau, 2013; Tang et al., 2011). However, with improvements in the handling process, the reliability of measurements can be achieved. Second, the sample size in our human study was small, and larger studies are required to confirm our findings.

In conclusion, CEUS is able to detect renal perfusion in a dynamic way. PI and TTP values could be used for the detection of renal microvascular damage in rats and patients with early-stage HN. Renal perfusion measured by CEUS correlates with renal functional impairment and tubulointerstitial injury and fibrosis in rats and patients with HN. These findings support the future clinical application of CEUS for the detection and diagnosis of HN in patients with hyperuricemia.

## MATERIALS AND METHODS

### Animal models

Male Sprague–Dawley rats weighing 180-200 g were purchased from the National Mode Animal Centre of Nanjing University (Nanjing, China), housed under a constant 12-h light–dark cycle at a temperature between 21°C and 23°C, and allowed free access to food and water. All animal experiments were approved by the Animal Care and Ethics Committee of the Sixth People's Hospital Affiliated with Shanghai Jiao Tong University and complied with relevant local animal welfare laws, guidelines and policies. The rats were randomly divided into four groups (*n*=8/group), and the HN rat model was induced by oral administration of a mixture of adenine (0.1 g/kg/day) and potassium oxonate (1.5 g/kg/day) daily for 4 weeks. The animals were sacrificed after 4 weeks.

### Patients

The research participants signed written informed consent forms. To further confirm the feasibility of CEUS in HN patients, renal cortical perfusion was measured by CEUS in 40 patients (mean age, 60±14 years; male) who were admitted for HN between October 2016 and April 2021. These patients all had a long history of hyperuricemia, recurrent attacks of gout and kidney stones detected by ultrasound. Hyperuricemia was defined as a fasting serum uric acid level higher than 420 μmol/l in men and 360 μmol/l in women (Sánchez-Lozada et al., 2007). CEUS was also performed in ten age- and sex-matched healthy volunteers (mean age, 58±12 years; male). CKD was diagnosed based on the Kidney Disease Outcomes Quality Initiative (KDOQI) classification (Yu et al., 2018). Patients with acute gout, acute kidney injury, interstitial nephritis, primarily glomerular nephritis, diabetic nephropathy or other secondary kidney diseases, and those who required maintenance dialysis or renal transplantation, were excluded. All the patients enrolled in this study had good blood pressure control. This study was approved by the institutional ethics committee of Shanghai Sixth People's Hospital on Human Research, 2020-KY-003(k). All clinical investigation was conducted according to the principles expressed in the Declaration of Helsinki.

### Ultrasound

#### CDUS, PDUS and PWD

Normal ultrasound was performed by one radiologist with 10 years of experience in kidney imaging using the Aplio 500 system (Toshiba Medical Systems Corporation, Tokyo, Japan) with a 7e18-MHz linear transducer. Two-dimensional ultrasound, CDUS and PDUS were included in the examination. The examination was carried out using the same machine parameters each time. Two-dimensional grayscale images of the kidneys were obtained, followed by evaluation of renal vascularity with CDUS, PDUS and PWD. We optimized the image depth, gain, focus and frame rate for each rat/patient during baseline and used identical parameters for further measurements.

#### CEUS

Microbubble-based contrast agents (MicroMarker, Bracco, Milan, Italy) were diluted in sterile 0.9% saline and injected into the rats through the tail vein using a syringe pump rate of 70-85 l/min. A destruction–reperfusion sequence was applied for CEUS. Nonlinear contrast images were obtained according to the theory of amplitude modulation. The low mechanical index imaging mode (mechanical index=0.15) was used until and 25 s after the contrast agent concentration reached the plateau state. A high mechanical index burst (mechanical index=1.6) was used to destroy the microbubbles. Images were recorded during microbubble infusion.

#### Ultrasound image analysis

Image analysis was performed with SonoLiver software 1.1 from TomTec Imaging Systems GmbH (Munich, Germany). The nonlinear contrast images were reversed to yield linearized intensity data. The data were baseline subtracted and normalized according to peak intensity. TTP (measured in s) was defined as the time required for the normalized intensity to reach 95% of the peak intensity. This was computed from a single TIC for each animal.

### Measurement of blood and urine biochemical indexes

Serum uric acid, creatinine, BUN and other biochemical indexes were measured by an automatic biochemistry analyzer (Model 7600, Hitachi, Tokyo, Japan). Serum KIM-1 was measured using an enzyme-linked immunosorbent assay (ELISA) kit (R&D Systems, Minneapolis, MN, USA).

### Histology and morphometry analysis

Tissues were either embedded in paraffin or frozen in optimal cutting temperature (OCT) compound and then sectioned to 3 μm thickness for light or polarized light microscopy. H&E and Masson trichrome staining were performed to assess histological injury and fibrosis. Frozen kidney sections were used to measure the anisotropism of uric acid crystals under a polarized light microscope. Quantification of MUCs was carried out using ImageJ software. A total of 20 views per slide were randomly obtained with a digital camera at 200× magnification. Crystal numbers and area were counted.

### Immunohistochemistry staining analysis

Formalin-fixed kidneys were embedded in paraffin. Kidney sections were stained using the following antibodies: anti-α-SMA (ab5694), anti-collagen 1 (ab34710) and anti-F4/80 (ab6640) (Abcam, Cambridge, MA, USA). The staining of the glomerulus and tubulointerstitium was semiquantitatively scored separately on a scale of 0-4 in a blinded manner by two independent researchers as previously described (Granata et al., 2011).

### Real-time PCR quantitation

Total RNA was extracted from renal cortex samples using TRIzol (Invitrogen, Carlsbad, CA, USA). RNA was reverse transcribed into cDNA using Superscript III First-Strand Synthesis Super Mix (Invitrogen). Real-time PCR was performed with a StepOne Plus System (Applied Biosystems, Foster City, CA, USA) using SYBR Green Master Mix (Qiagen, Hilden, Germany). Using the 2−ΔΔCt method, relative gene expression levels were calculated. Gene expression was normalized to that of *Gapdh*.

### Apoptosis assessment

Apoptotic cells were measured in frozen kidney sections using an ApopTag R Red In Situ Apoptosis Detection Kit (Millipore, Billerica, MA, USA). The number of apoptotic cells with nuclei stained with red fluorescence was calculated using fluorescence microscopy. Ten fields per slide were quantified for apoptotic nuclei.

### Statistical analysis

Data were analyzed with SPSS software 19.0 (IBM, Armonk, NY, USA) and are presented as mean±s.e.m. Correlations were assessed according to Pearson's correlation analysis. One-way ANOVA followed by Bonferroni correction was used to compare the data of more than two groups, while a unpaired, two-tailed *t*-test was used for nonparametric data comparison. A value of *P*<0.05 was considered statistically significant.

## Supplementary Material

Supplementary information
