# Supplementary material for: Evaluation of renal microperfusion in hyperuricemic nephropathy by contrast-enhanced ultrasound imaging
Source: Dis Model Mech. 2022 Jul 26;15(7):dmm049382. doi: 10.1242/dmm.049382 (PMC9346517; doi:10.1242/dmm.049382)
Supplement: Supplementary information [file dmm-15-049382-s1.pdf]

Table S1: Kidney injury in rats with mild and severe HN

Variables	Control	HN 1w	HN 2w	HN 4w
ALB(g/L)	39.00±2.00	37.67±1.15	37.20±3.27	40.20±1.92
BUN(mmol/L)	5.36±0.32	8.65±0.78*	12.08±1.63**	13.80±2.77**
Scr(μmol/l)	19.91±1.70	29.33±2.08**	38.80±5.45**	49.00±6.78**
UA(umol/L)	61.50±10.82	104.33±19.63*	117.75±24.68**	195.00±31.75**
ALT(u/L)	40.67±6.28	49.33±1.15	43.80±8.93	47.00±6.68
AST(u/L)	161.40±13.07	167.50±14.85	178.50±13.44	167.00±10.54
TC(mmol/L)	1.33±0.16	1.29±0.13	1.14±0.42	1.99±0.16**
TG(mmol/L)	0.94±0.34	0.67±0.52	0.59±0.11	0.80±0.48
HDL(mmol/L)	0.69±0.09	0.65±0.04	0.69±0.19	0.67±0.07
LDL(mmol/L)	0.20±0.04	0.22±0.03	0.18±0.05	0.23±0.08
BG(mmol/L)	7.54±0.92	6.79±0.63	5.41±3.16	6.42±3.71
UACR (mg/mmol)	3.70±1.15	16.36±4.31**	23.56±2.1**	25.23±3.32**
SBP(mmHg)	115.0±3.64	125.8±2.64**	135.5±4.03**	145.8±1.30**
Kidey/BW(mg/g)	2.76±0.15	3.85±0.24*	4.29±0.31**	5.53±0.29**

Abbreviations: HN: hyperuricemic nephropathy; BUN: blood urea nitrogen ; TC: total cholesterol ; TG: triglyceride; ALB: serum albumin; ALT: Alanine transaminase ; AST: aspertate aminotransferase ; HDL: high-density lipoprotein; LDL: low density lipoprotein ; BG: blood glucose ; and UACR: urine albumin-to-creatinine ratio ; BW: Body Weight; *$p<0.05$, **$p<0.01$ for comparisons between the corresponding HN and control groups;

Disease Models & Mechanisms • Supplementary information
